# Outcome of modified interlaminar decompression: A conservative decompressive surgery for lumbar spine stenosis

**DOI:** 10.12669/pjms.36.4.1170

**Published:** 2020

**Authors:** Farooq Azam, Seema Sharafat, Zahid Khan, Mumtaz Ali

**Affiliations:** 1 Farooq Azam, Department of Neurosurgery Medical and Teaching Institute, Lady Reading Hospital, Peshawar, Pakistan; 2 Seema Sharafat, Department of Neurosurgery Medical and Teaching Institute, Lady Reading Hospital, Peshawar, Pakistan; 3 Zahid Khan, Department of Neurosurgery Medical and Teaching Institute, Lady Reading Hospital, Peshawar, Pakistan; 4 Mumtaz Ali, Department of Neurosurgery Medical and Teaching Institute, Lady Reading Hospital, Peshawar, Pakistan

**Keywords:** Lumbar Spine, Lumbar Spinal Stenosis, Laminectomy, Lumbar Decompression, Lumbar Disc Herniation, Lumbar Fusion

## Abstract

**Objective:**

To evaluate the outcomes of modified interlaminar decompression in patients with degenerative lumbar spinal stenosis (LSS).

**Methods:**

This descriptive observational study was conducted at the Department of Neurosurgery, Lady Reading Hospital Peshawar from July 2014 to June 2018. All patients with degenerative LSS who underwent modified interlaminar decompression during the study period were included in the study. The patients were followed up to one year after surgery. The data was entered into a structured questionnaire designed according to the study which was then analyzed using SPSS version 21.

**Results:**

A total of 182 LSS cases were included in the study and 236 levels were operated during the study period. According to the records increased prevalence of LSS was found among males i.e. 58.8%. The common level with degenerative stenosis involved was L4-5. Good to excellent outcomes were observed in 93.9% patients in the 1^st^ follow-up visit. The most common complication of surgery was dural tear followed by wound infection.

**Conclusion:**

Modified interlaminar decompression is a conservative surgical technique, proved to be a potential approach with acceptable complications, satisfactory outcomes and it is easy to learn.

## INTRODUCTION

Lumbar stenosis is one of the most common spinal pathologies resulting in the abnormal narrowing of the spinal canal and intervertebral foremen leading to degeneration.^1^ Neurogenic pain, lower back pain, numbness and recurrent cramps are commonly observed clinical complaints among patients with LSS. Moreover, gait disorder and cauda equine syndrome (leg weakness and sphincteric dysfunction) are also associated with lumbar stenosis.^2^

LSS could either be congenital or acquired, where the congenital form mostly occurs in lumbar tract and is present by birth.^3^ It is associated with alterations in the pedicles, changes in the sagittal orientation of the facet joints and shortness of laminae which further reduces the size of the spinal canal. While the acquired form is commonly observed in both cervical and lumbar tracts, among the narrowing factors hypertrophied ligamentum flavum, intervertebral disc herniation, hypertrophied facet joint and spondylolisthesis had been reported significantly.^4^ Systematic illnesses might play a role in the etiology of acquired LSS i.e. inflammatory, infectious diseases, metabolic disorders (calcium) and endocrinopathies are the common co-morbid conditions associated with LSS.^4^

There is no gold standard diagnostic procedure recommended by the physicians for LSS patients however, patient history, physical examination, and neuroimaging had been widely used for diagnosis.^5^ The physical factors that are most likely to be associated with LSS and must be considered during diagnosis include pain due to lumbar flexion, age, neurogenic claudication, abnormal gait and irregular Romberg test results.^2^,^4^ For radiographic examination plain radiography, magnetic resonance imaging (MRI), computed tomography (CT) scan and myelography had been frequently utilized.^6^ Other rarely used diagnostic techniques include electromyographic technique-paraspinal mapping, selective lumbar nerve root block, magnetic stimulation caudal motor conduction time, dermatomal somatosensory evoked potentials but their accuracy remains uncertain.^7^

Numerous treatment modalities had been discovered and utilized for treating LSS, the non-operative procedures include medications, epidural injections, lifestyle modifications and multidisciplinary rehabilitation but there is lack of data supporting the effectiveness of non-operative methods.^8^ Surgical interventions including decompression, interspinous spacers and lumbar fusion are recommended for the cases where the symptoms persist.

The treatment efficacy of LSS either with decompression together with fusion or decompression alone has been a point of debate and the controversy still continue. Fusion in comparison to decompression is a complex intervention and increases post-operative complications, mortality rate and the treatment cost.^9^ Therefore, modified inter-laminar decompression is an attractive treatment option to date, as it preserves most of the posterior elements and only ligamentum flavum is resected and under-cutting of the facet joint is done upon requirement.^10^ This procedure has the benefit to decompress the neural elements without compromising stability and motion of the spine and patient can return back to normal routine much earlier as it is expected with other procedures.^10^

Locally not much data is available regarding modified interlaminar decompression for LSS. Therefore, though this study our aim was to discover the effectiveness of the decompressive procedure for LSS patients under local clinical setting.

## METHODS

### Study Design, Duration & Setting

This descriptive observational study was conducted at the Department of Neurosurgery, Lady Reading Hospital Peshawar from July 2014 to June 2018. A total of 182 patients with degenerative LSS irrespective of their age and gender were included in the study. Moreover, only those LSS patients who underwent modified interlaminar decompression during the study period were recruited while patients with recurrent lumbar stenosis treated conservatively or underwent conventional laminectomy were excluded from the study sample.

### Surgical procedure

Under general anesthesia patient is rested in prone position. Dorso-lumbar fascia is incised preserving the supra-spinous ligamentous. Unilateral muscle is stripped from the spinous and laminar attachments, up to medial portion of the facet joint. The spinous process of the above vertebra at the involved segment is fractured at the spino-laminar junction with curved osteotome or bone nibbler. Suppose when L3/L4 spinal canal stenosis is decompressed, the L3 spinous process is fractured at spino-laminar junction. Self-retaining retractor is applied to expose the segment to be decompressed. The stripping and retraction of paraspinal musculature should not be extend to the facet joint on the opposite side as well. Minimal bony resection of the lamina above and below at the involved segment is done to release ligamentum flavum from its above, below, medial and lateral attachments. For multiple levels same procedure is repeated so that bony bars of the remaining lamina are left. Decompression of the dural sac and the nerve roots is done. The retractor is removed and the osteotomized spinous process resumes its position in contact with retained bar (portion) of lamina. Hemostasis is confirmed, the dorso-lumbar fascia is repaired with vicryl size 1.

### Ethical Concerns & statistical analysis

After receiving ethical approval from Lady Reading Hospital MTI (Reference no. 130/LRH; Dated: May 20^th^ 2013), informed consent was taken from each patient or their relatives. Medical records of the patients including demographic data, clinical features, neuroimaging details were used for assessment. Magnetic resonant imaging (MRI) was used for the confirmation of pre-diagnosed LSS among the study subjects. The patients were followed up to one year after surgery. Clinical and functional outcomes of the surgery were assessed based on the Stucki’s Criteria. The extracted data was utilized to fill in the study structured questionnaire designed for the study. Data was analyzed using Statistical Package for the Social Sciences (SPSS) version 21. Variables are denoted as frequency and percentages.

## RESULTS

Out of the total 182 patients 58.8% (107/182) were males while the rest 41.2% (75/182) were females. Minimum time of surgery recorded was 26 minutes (single level) and maximum time was 83 minutes (multiple level) with average time of 51 minutes. A total of 236 levels were operated among 182 patients during the study periods and the most common level of surgery was L4–5 followed by L3–4 level.

According to the Stucki’s Criteria, much of the cases had good surgery outcomes and only 1.1% were in the poor category in the 1^st^ follow up visit as shown in Table-III. The outcomes were similar in the 2^nd^ and 3^rd^ follow-up visit.

**Table-I:** Clinical and functional outcome.

Outcomes	Condition after surgery
Good to Excellent	Occasional mild back and leg pain. Ambulatory distance more than one mile or 20 minutes. No restrictions in usual activities.
Fair	Persistent mild back or leg pain with occasional moderate pain. Less than one mile or 20 minutes of ambulation. Mild restrictions in usual activities.
Poor	Little to no pain relief from surgery. A repeat operation for any reason was considered a poor result. Major activity limitations

* Stucki et al., criteria for assessment-Table-I.

**Table-II:** Surgical details of the study subjects.

Lumbar level of surgery	n(%)
L2-3	7(3.8)
L3-4	32(17.6)
L4-5	76(41.7)
L5-S1	22(12.1)
Multiple level (up to 3 level) 2 levels 3 levels	45(24.7) 36(80) 9(20)

*n=182; total levels= 236

**Table-III:** Outcomes of surgery on the basis of Stucki Criteria.

Surgery Outcomes	Visits		

	1^st^ Follow Up	2^nd^ Follow Up	3^rd^ Follow Up
Good /Excellent	171(93.9)	168(92.3)	166(91.2)
Fair	9(5)	12(6.6)	14(7.7)
Poor	2(1.1)	--	--

*values are given as n(%).

**Table-IV:** Surgical complications observed in the study population.

Complications	Visits		

	1^st^ Follow Up	2^nd^ Follow Up	3^rd^ Follow Up
Dural tear	6(3.3)	--	--
CSF leak	1(0.6)	--	--
Wound Infection	3(1.6)	--	--
Neurodeficit	1(0.6)	--	--
Segmental Instability	--	--	--
Reoperation	--	--	--

*CSF = Cerebrospinal Fluid; Values are given as n(%).

Dural Tear was the most reported complication followed by wound infection in the first follow-up visit. Complications were also monitored during the 2^nd^ and 3^rd^ follow up visits but none appeared.

## DISCUSSION

In our study LSS was found more commonly among males as compared to females. In contrast a study report increased prevalence of LSS in females.^11^ Literature suggest that age, sex and body mass index (BMI) are among the common physiological parameters that increases the risk of surgical decompression, where smoking and drinking habits further precipitate the condition.^12^-^14^ Our study presented more young patient with LSS in comparison to the older counterparts. A total of 236 lumbar levels of surgery with modified interlaminar decompression were operated in the study patients, out of which L4-L5 was the common lumbar level operated, similarly previous finding also indicated that at L4-L5 level, lumbar stenosis disc space narrowing (DSN) was most common.^14^

Treatment outcomes and effectiveness indicated by the previous studies suggest that surgical decompression remains the intervention of choice^15^, where conventional laminectomy, unilateral laminotomy, bilateral laminotomy, partial facetectomy and split-spinous process laminotomy/laminoplasty are commonly used surgical interventions.^16^ Moreover, interspinous spacer is an alternative treatment for LSS, it is minimally invasive and can be implanted percutaneously with open or microsurgical decompression or alone without decompression.^17^ Modified endoscopic interlaminar decompression technique have recently been popularized for lumbar stenosis^19^, studies reported excellent results among majority of the patients treated with microendoscopic decompression.^18^,^19^ Our findings also supported the fact, 91.2% of the patients were observed with good to excellent outcomes after surgery along with 6.6% of the complication rate therefore indicating a success rate of 90%. Among the post-operative complications, dural tear were observed among our study patients followed by wound infections while no cases of re-operation were reported. In contrast, no cases with dural tear were reported in study conducted in Japan on lumbar stenosis patients who underwent microendoscopic decompression surgery while they reported only 2 cases with spinous fractures.^20^

On the basis of operative complications associated with other decompressive surgeries, it is evident that modified interlaminar decompression is comparatively safer. In relation, a systematic review revealed that surgical decompression techniques for LSS show greater benefit as compared to other techniques including interspinous spacer devices and fusion etc.^21^ Excessive intraoperative blood loss is observed among the LSS patients treated with decompression along with fusion.^21^ As far as the duration is concerned interspinous spacers revealed shorter operative duration as compared to other techniques,^22^ but its use under the local clinical setting is debatable as these devices are expensive and require surgical revisions. High quality trials are required in order to identify the treatment modality for LSS with maximum efficacy and overall cost effectiveness. So that the best surgical option for this condition can be ruled out.

Although, the study results revealed good outcomes associated with modified interlaminar decompression technique among LSS patients but there were some limitations of the study. The outcomes of modified interlaminar decompression were identified but the effectiveness was not monitored in comparison to any other technique. Moreover, the study was single-centre, such estimation must be conducted on larger scale including multiple centres for more significant results indicating the local population.

## CONCLUSION

Minimally invasive decompression and non-fusion techniques are effective for the treatment of LSS with comparatively low reoperation rates. Although the clinical outcomes are comparable but this conservative surgical technique lowers the instability rate after procedure. Moreover, the benefits of minimally invasive surgery cannot be neglected such as decreased blood loss and shorter hospital stay. Keeping all the potential benefits associated with minimally invasive modified interlaminar decompression technique, the necessity for other fusion extensive surgical procedure must be re-evaluated.

### Authors’ Contribution

**FA** is responsible for the concept and is responsible for integrity of research.

**SS** contributed to the data collection and literature review.

**ZK** is responsible for data analysis and interpretation and drafting of the manuscript.

**MA** contributed to the concept and critical revision of the study.
